# Gender roles and traits in stress and health

**DOI:** 10.3389/fpsyg.2015.00779

**Published:** 2015-06-09

**Authors:** Eric Mayor

**Affiliations:** Institut de Psychologie du Travail et des Organisations, Université de Neuchâtel, Neuchâtel, Switzerland

**Keywords:** inequity in health, stress, cognitive appraisal, gender, masculinity, femininity

## Abstract

Women have a life-expectancy advantage over men, but a marked disadvantage with regards to morbidity. This is known as the female–male health-survival paradox in disciplines such as medicine, medical sociology, and epidemiology. Individual differences in physical and mental health are further notably explained by the degree of stress individuals endure, with women being more affected by stressors than men. Here, we briefly examine the literature on women’s disadvantage in health and stress. Beyond biological considerations, we follow with socio-cognitive explanations of gender differences in health and stress. We show that gender roles and traits (masculinity in particular) explain part of the gender differences in stress, notably cognitive appraisal and coping. Stress in turn degrades health. Implications are discussed. In conclusion, traditional socialization is advantageous for men in terms of health.

## Introduction

Women endure higher rates of physical and mental diseases than men at all ages and in all regions of the world (Macintyre et al., 1996; Austad, 2006; Seedat et al., 2009). They experience more days of health-related reduced activity and work cessation, higher rates of hospital stays, and have higher health care costs, even when excluding diseases of the reproductive system (Wingard et al., 1989; Green and Pope, 1999; Case and Paxson, 2005). Women’s higher healthcare use further reduces their economic resources (Green and Pope, 1999; Bertakis et al., 2000; McIntyre et al., 2006). It is therefore essential to better understand gender differences in health.

Here we propose socio-cognitive explanations of gender differences in health. As stress impacts health considerably, we also focus on gender differences in stress and their explanations. We will show in particular that social roles and gender traits explain part of the observed gender differences in health and stress. Below, we start by examining the magnitude of gender differences in health.

## Magnitude of Gender Differences in Health

**FIGURE 1:**
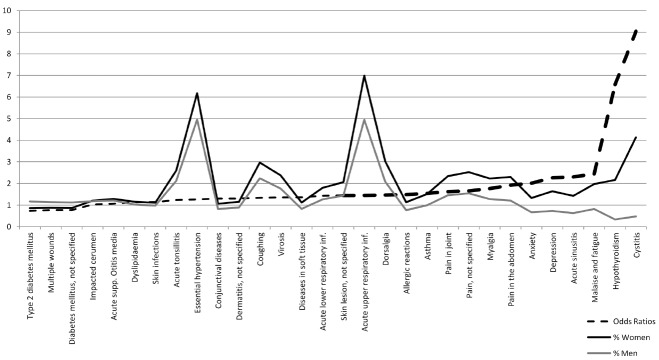
**Most frequent diagnostic groups in primary care**.

Among the 30 most common diagnostic groups in primary care, 90% are more common in women than in men (*N* = 2 mio, Stockholm; Wändell et al., 2013). The median of odds ratios (ORs) among the 30 groups of diagnoses is 1.43, i.e., the odds of a diagnostic group being applied to women are 143% that of men, or higher, for half of the 30 diagnostic groups. These 15 diagnostic groups represent 56% of individual diagnoses analyzed. Figure 1 presents the ORs and percentage of women and men affected. Note these results are not based on self-reports, but official statistics.

Most primary care diagnostics are related to acute conditions. Similar relationships are observed in chronic conditions: The total number of chronic conditions reported by individuals is at least 40% higher for women than for men (Canadian National Health Survey; see Quesnel-Vallée et al., 2008). Further, such gender differences exist throughout adulthood (US National Health Interview Survey; Verbrugge, 1986). Overall, ORs (women:men) are 1.25 for longstanding illness, 1.17 for limiting longstanding illness, and 1.92 for somatic symptoms (*N* = 17,300; controlling for age; Lahelma et al., 1999).

## Potential Bias in Self-Report Data

Findings based on self-reports might be affected by the ability of individuals to report health information (e.g., over-reporting by women) and the impact of self-selection bias. Over-reporting is not empirically supported for the majority of diseases. Research shows that women and men are equally accurate at reporting health information, especially for chronic conditions (e.g., Cleary and Jette, 1984; Macintyre et al., 1999; Ritter et al., 2001). But a few diseases feature gender differences (in both directions) in the quality of reporting (Oksuzyan et al., 2009). Participation rates are slightly higher among hospitalized women compared to those who are not, whereas hospitalized men do not differ (*N* = 15,330; Oksuzyan et al., 2009). This excess in participation of sicker women could contribute marginally to gender differences. But in the elderly, participation rates are higher among men (*N* = 1,045; Jacomb et al., 2002). As health declines with age, gender differences in health might therefore be higher than those reported in overall estimates.

## The Impact of the Environment

Individuals’ behavior, their social environment, and genetic factors can cause women’s health disadvantage (Oksuzyan et al., 2010). Yet, the genetic code interacts with the environment to produce changes between and within individuals that are relevant for health (e.g., Feinberg, 2007): the environment as well as environmental variations are known to affect respectively phenotypes (individual’s physical stable characteristics) and gene expression—the moment-by-moment translation of genes into RNA, proteins and hormones, allowing rapid adaptation to changing conditions (Dunn and Plomin, 1990; Jaenisch and Bird, 2003; DeWitt and Scheiner, 2004).

In the social sciences, research on intersectionality and health further discusses the caveats of focusing solely on sex when discussing health differences between women and men (see Hankivsky, 2012): sociodemographic factors also have to be considered. Gender differences in a number of physical and mental diseases, and functional health, disappear when controlling for socio-demographic and contextual factors (*N* = 1,641; Emslie et al., 1999; *N* = 3,522; Quesnel-Vallée et al., 2008), but not differences in the number of chronic conditions (Quesnel-Vallée et al., 2008).

## Gender, Stress, and Health

The environment explains part of the gender differences in health, and this might be mediated by stress. Indeed, women and men differ in their exposure and reactions to stressors (Lazarus and Folkman, 1984; Kudielka et al., 2007): women experience more chronic stressors than men (McDonough and Walters, 2001; Matud, 2004) and consider stressors as more threatening (Lazarus and Folkman, 1984; Ptacek et al., 1992).

Stress activates the hypothalamic-pituitary-adrenocortical axis and the sympathetic-adrenal-medullary axis, leading to the release of stress hormones (e.g., cortisol and catecholamines) that trigger dysfunctions of the immune system related to various diseases (Frankenhaeuser, 1986; Ader and Cohen, 1993; Segerstrom and Miller, 2004; Glaser and Kiecolt-Glaser, 2005; Lundberg, 2005; Cohen et al., 2007). Here we describe part of this process in a nutshell. On the one hand, cortisol secretion decreases lymphocytes counts (notably natural killer cells), which results in increased vulnerability to diseases and infections (Gatti et al., 1987; Padgett and Glaser, 2003). On the other hand, catecholamines induce an elevation of pro-inflammatory cytokines (Johnson et al., 2005). Abnormally high concentrations of pro-inflammatory cytokines are related to several conditions and risks including cardiovascular risk factors and coronary heart disease, diabetes mellitus, inflammatory and autoimmune diseases, depression and schizophrenia (Lucey et al., 1996; Rabinovitch and Suarez-Pinzon, 1998; Blanco et al., 2008; Miller et al., 2009; Watanabe et al., 2010).

Indeed, psychological stress predicts a variety of somatic diseases and mental illnesses (Steptoe, 1991; Cohen et al., 2007). Stressed individuals are more at risk of developing depression and psychosis (Cohen et al., 2007; Stroud et al., 2008; van Winkel et al., 2008). Somatic complaints related to stress can range from minor or mild symptoms and diseases such as sore throat, headaches and influenza, to life threatening conditions such as heart attacks, cancer, and organ failure (Selye, 1976; DeLongis et al., 1988; Kiecolt-Glaser et al., 1992; Reiche et al., 2004; Cohen et al., 2007). Further, chronic diseases can be triggered by high levels of stress (Kendall-Tackett, 2010). In particular, the onset of autoimmune diseases is preceded by important stress episodes in 80% of cases (Herrmann et al., 2000; Stojanovich and Marisavljevich, 2008). Differentiated exposure to stressors explains part of the gender differences in health (McDonough and Walters, 2001).

## Gender Roles, Illness, and Stress

Among the many social explanations for the differences between women and men regarding health, those related to socio-economic status and social roles have received most empirical support (Link and Phelan, 1995). Indeed, some if not most of the non-physiological differences between women and men are socially constructed rather than biologically determined. In the (psycho)sociological literature, the term “sex” refers to the genetic and physiological differences between women and men, whereas the term “gender” refers to the experience of being male or female and the traditionally differentiated social roles they accomplish (e.g., home makers vs. resource providers—hereafter gender roles; Deaux, 1985; Eagly et al., 2000). In their biosocial constructionist theory, Eagly and Wood (2013) describe how social expectations and biological processes (e.g., men not being able to give birth) interact to maintain such roles, but also allow for change. Differentiated social roles are important factors in the higher morbidity observed in women (Oksuzyan et al., 2010), primarily because of the differentiated stressors and stress reaction they induce (Lundberg, 2005). Below we present some of the empirical findings regarding the impact of social roles on health.

Women who work, compared to those who do not, face less physical and mental health problems (Natalson, 1980; Rosenfield, 1980; Kessler and McRae, 1982; Repetti et al., 1989): providing care, particularly to family members (the “caregiver health effect”; Schulz and Beach, 1999) negatively impact both physical and mental health (e.g., Kessler et al., 1985; Schulz et al., 1989). This is related to the higher number of stressors affecting caregivers, compared to non-caregivers (Son et al., 2007). Because women are more often caregivers than men, their risk of exposure to such strain is higher, and this impacts their health (Repetti et al., 1989; Eagly et al., 2000; Son et al., 2007).

As the participation of women in the workforce has increased, stress differences between women and men have decreased (Jick and Mitz, 1985). Women often also occupy clerical positions, whereas men occupy leadership positions more often than women (Eagly et al., 2000). High-status individuals have more control, which leads to less experienced stress, whereas lower-status individuals experience more stress and use less efficient coping strategies (Thoits, 1991; Bakker and Demerouti, 2007).

When working under similar conditions, women and men do not differ in their release of stress hormones, heart rate and experience of stress, whereas they do otherwise (Persson et al., 2009). Further, the excess of occupational stress of women compared to men disappears when age, education, and marital status are accounted for (*N* = 2,775; Galanakis et al., 2009). With the increased similarity in the gender roles over the years, women’s physiological responses become more similar to those of men (Frankenhaeuser, 1991).

## Gender Traits (Masculinity/Femininity), Illness, and Stress

Throughout life, people are taught (explicitly, implicitly, and vicariously) which behaviors are desirable for men or women in society (Bem, 1981). Examples include experiencing that women usually take care of children while men work, the encouragement to participate in gender typical activities, or injunctions (for children: “boys don’t cry,” “girls don’t swear”). At a later age, such expectations often translate in the approval and disapproval of conduct (see Bussey and Bandura, 1999; Martin et al., 2002).

Behavioral expectations are integrated by individuals into their identity (gender traits), and the typicality of gender traits (masculinity and femininity) follows their desirability (Bem, 1981; Prentice and Carranza, 2002). Masculinity refers to the traditional behavioral expectations for men (Bem, 1981). Femininity refers to the traditional behavioral expectations for women. Masculinity, also referred to as instrumentality or agency; and femininity, also referred to as communion and emotionality (e.g., Colley et al., 2009), are usually measured using the Bem Sex Role Inventory (Bem, 1981) and the Personal Attributes Questionnaire (Spence et al., 1974). These are questionnaires where participants rate themselves on items such as dominant, aggressive, assertive and independent for masculinity; and caring, gentle, compassionate and emotional for femininity.

Research on the relationship between gender traits and health has shown that masculinity in particular is related to better physical and mental health (controlling for gender). Masculine individuals have better self-assessed general health, less physical symptoms and better mental health, and consult general practitioners less often (*N* = 886; Annandale and Hunt, 1990). Masculinity is positively related to physical health and health behaviors (*N* = 336; Shifren and Bauserman, 1996). Overall, the effect of masculinity on physical health is positive, except for coronary heart disease (explaining in part the higher mortality of men; Helgeson, 1994; Barrett-Connor, 1997). With regard to mental health, masculinity is negatively related to anxiety, tiredness, and depression, but to higher antisocial behavior and substance abuse (Emslie et al., 1999; Lengua and Stormshak, 2000). A meta-analysis of 26 studies (Bassoff and Glass, 1982) shows that masculinity is robustly associated with better mental health. Femininity is also positively related to health promoting behaviors (Shifren and Bauserman, 1996). But the research shows conflicting findings in the relationship between femininity and health. Femininity is related to higher risks of depression in women (Tinsley et al., 1984; Bromberger and Matthews, 1996), and to better and worse physical health outcomes depending on the studies (Shifren and Bauserman, 1996; Hunt et al., 2007).

### Cognitive Appraisal

Stressors can be appraised as a threats or challenges (cognitive appraisal). Threat appraisals occur when resources (e.g., knowledge, skills, money) are considered insufficient to face a stressor. Challenge appraisals occur when individuals perceive they might benefit from the encounter with the stressor and consider their resources sufficient. Cognitive appraisals mediate the relationship between stressors and outcomes such as wellbeing and health (Lazarus and Folkman, 1987). In particular, threat appraisals are negatively related to physical health and positively to psychological symptoms (Folkman et al., 1986b). Threat appraisals are related to physiological responses that promote exhaustion and disease, such as an excess in the liberation of catecholamines and cortisol, whereas challenge appraisals have less damaging outcomes and promote productivity and focus (Frankenhaeuser, 1986; Benschop et al., 1996).

An emerging body of research focuses on the relationships between gender traits and cognitive appraisals (e.g., Sarrasin et al., 2014). Highly assertive women exhibit the physiological responses of a challenge appraisal and perform better than less assertive women, who have patterns of physiological measures consistent with threat appraisal (Tomaka et al., 1999). Surveys feature similar results (Long et al., 1992; Sarrasin et al., 2014). A study with two different samples (for replication purposes; N1 = 322, N2 = 525; Sarrasin et al., 2014) shows that masculinity is robustly and positively related to challenge appraisal in both women and men. Masculinity is also negatively related to threat appraisal in men in both samples, and women in the second sample.

The relationship between femininity and cognitive appraisal is not so robust, in line with its impact on health outcomes. Gender might moderate the impact of femininity on cognitive appraisal. Femininity increases the threat appraisal of several health risks in men, but decreases threat appraisal in women (Kaplan and Marks, 1995). Sarrasin et al. (2014) report that femininity is positively related to challenge appraisal only in women of the second sample.^1^

### Coping

Gender traits also predict coping mechanisms. There are two families of ways of coping: problem-focused (such as active coping, planful problem-solving) and emotion-focused (e.g., avoidance, disengagement; Folkman et al., 1986a). Problem-focused coping is related to better health outcomes (Folkman et al., 1986b). Masculine traits are positively related to active coping, and negatively to avoidance coping (Lengua and Stormshak, 2000). The role of femininity is less clear as it has been found to be positively and negatively related to active coping (Lengua and Stormshak, 2000; Gianakos, 2002). Masculinity and femininity are related to seeking social support and positive thinking (Gianakos, 2000, 2002). Masculinity is indirectly linked to distress in the face of stressors (negatively) through challenge appraisal and disengagement coping (Long et al., 1992).

## Discussion and Conclusion

Even though men perform less health-related behaviors than women (Deeks et al., 2009), they are less at risk to contract a large majority of health conditions. Our socio-cognitive perspective on gender inequity in health contributes to the current debate (see Doyal, 2000). Gender differences in health and stress have been described. Socialization, particularly through gender roles and gender traits, has been related to the stress process, the experience of stress, and to the health of individuals. We have shown that gender traits have implications with regards to the way stressors are dealt with, and ultimately morbidity. In particular, we have shown that gender traits are related to the appraisals of stressors (as threats or challenges), which play an important role in health.

The literature reviewed here shows that masculinity is beneficial for health. Nevertheless, untempered masculinity comes with its own risks with regards to health and mortality (Helgeson, 1994; Barrett-Connor, 1997; Lozano et al., 2012). Masculinity encourages aggressive conduct and the misperception of its consequences (Eagly and Steffen, 1986). Around the world, 62% of the individuals who died in 2010 at ages 15–49 were men (Global Burden of Disease study; Lozano et al., 2012). Causes of death related to the behavior of self and others (mostly violence from others or from self) represent 38.3% of the deaths in men in this age range, against 23.5% of the deaths in women. Causes of death that involve physical diseases account for 76.6% of women’s deaths, against 61.6% of men’s deaths. Higher rates of behavior- and aggression-related causes of death in men are part of the explanation for the difference in mortality between women and men.

The socialization of women and men remains quite differentiated. Women are still expected to be more nurturing and emotional (feminine) than men, who are expected to be dominant and instrumental in their conduct (masculine; Prentice and Carranza, 2002). Yet, it is notable that changing gender roles, such as the increased participation of women in the professional arena, have already decreased the higher morbidity of women and their higher rate of stress manifestations compared to men (Kessler and McRae, 1982; Jick and Mitz, 1985; Repetti et al., 1989; Matud, 2004). Yet, the conditions required for masculinity to be beneficial still need to be more fully identified: recent research also shows that when women are “too masculine” in society (with regards to social expectations), the overall health of men and women diminishes (Backhans et al., 2007), meaning that the health benefits of equality might only be truly reached after social expectations are changed. Egalitarian legislations are not enough.

Taken together, the literature reviewed here supports the proposition that traditional socialization is advantageous for men not only in terms of power, including economic resources (see Eagly et al., 2000), but also in terms of health, including exposure and reactions to stressors (Emslie et al., 1999; Matud, 2004; Sarrasin et al., 2014). The reasons for the maintenance of gender-based status asymmetries and their consequences are discussed in the edited volume “Gender and social hierarchies” (Faniko et al., in press), among other sources.

### Conflict of Interest Statement

The author declares that the research was conducted in the absence of any commercial or financial relationships that could be construed as a potential conflict of interest.

## Footnotes

1 Differences between the two samples exemplify the need for replication studies and meta-analyses (see Cumming, 2008). In the social sciences, and psychology in particular, the rate of results reproducibility might be 50%, whereas some fields of the medical sciences feature reproducibility rates between 11 and 25% (see Open Science Collaboration, 2012).
